# Chinese White Wax Solid Lipid Nanoparticles as a Novel Nanocarrier of Curcumin for Inhibiting the Formation of *Staphylococcus aureus* Biofilms

**DOI:** 10.3390/nano9050763

**Published:** 2019-05-17

**Authors:** Lin Luan, Zhe Chi, Chenguang Liu

**Affiliations:** College of Marine Life Sciences, Ocean University of China, Qingdao 266003, China; luanlin_ouc@163.com

**Keywords:** Chinese white wax, solid lipid nanoparticles, curcumin, biofilms, *Staphylococcus aureus*

## Abstract

Chinese white wax solid lipid nanoparticles (cwSLNs) were prepared by high shear homogenization and ultrasound methods. Using an optimized formula, spherical cwSLNs with an average particle size of 401.9 ± 21.3 nm were obtained. The cwSLNs showed high entrapment efficiency, approximately 84.6%, for loading curcumin. The curcumin loaded cwSLNs (Cur-cwSLNs) exhibited sustained drug release properties. Notably, Cur-cwSLNs had a higher drug release rate at pH 4.5 than at pH 7.4, which suggested their applicability in an acidic environment. Cur-cwSLNs were able to inhibit the growth of *Staphylococcus aureus* and were more effective at reducing the biofilms produced by this bacterium compared to free curcumin. This study confirmed that cwSLNs may be novel carriers for increasing the bioavailability of curcumin with the potential to inhibit the formation of *S. aureus* biofilms.

## 1. Introduction

Biofilm refers to a community of microorganisms that attach to a surface or group of microbial cells forming an aggregate [1]. In nature, almost 90% of microorganisms are able to form biofilms [2]. The bacteria in biofilm form are protected by extracellular polymeric substances (EPSs). EPSs work as a protective barrier, which contributes to the lower sensitivity and higher resistance of biofilms to antibiotics [3]. Consequently, EPSs allow biofilms to be about 1000 times more resistant to antibiotics than planktonic cells [4]. Intercellular interactions have also been observed in biofilms, leading to a high level of antibiotic resistance [5].

Presently, nanotechnology has provided many nanoparticle-based solutions for improving the antimicrobial effects of antibiotics for treating microbial infections caused by biofilms [6,7]. Of these nanoparticles, it is well-documented that solid lipid nanoparticles (SLNs) show certain colloidal carrier properties [7]. They have attracted much research attention because of their high stability over long periods and their high biocompatibility [6]. In particular, compared with glyceride SLNs, wax-comprised SLNs showed outstanding distribution of particle size and long-term physical stability [8]. However, it appears that the exploitation of wax-SLNs is just reaching its threshold. A natural and biological wax, Chinese white wax, which is produced by *Ericerus pela*, has been widely used in traditional medicine in China for about 1000 years. Chinese white wax comprises cerylcerotate, its major component, the constituents of C26–C30 straight-chain, unsaturated fatty acids, and their corresponding fatty alcohols, such as hexacosanol and octacosanol [9]. Nowadays, although a number of waxes can replace Chinese white wax, it is still widely used in pharmaceutical production, e.g., in pill coating and for sealing medicine bottles to prevent denaturation of the drug during storage [10]. The melting point of Chinese white wax (83–86 °C) is higher than that of many other waxes, such as the widely used paraffin wax (50–60 °C), and this property is suited to the preparation of more stable SLNs.

On the other hand, the use of SLNs represents a new perspective in the nanoparticle-based delivery of novel anti-infectives. The development of these new anti-infectives is due to the serious threat of increasing numbers of multiple drug-resistant pathogens that even hide beneath the structure of their biofilms. Medicinal plants provide an important source of many antimicrobial agents and powerful drugs [11,12], in which many active products able to fight against biofilms have been found. These include anthocyanin malvidin, tannins, terpenoids, and curcumin [13]. Curcumin is a natural polyphenolic compound that is extracted from the rhizomes of *Curcuma longa* [14]. It exhibits various biological activities, including antioxidant, antitumor, antimicrobial, and anti-inflammatory activity, as well as wound healing ability [15]. Although the antimicrobial and anticancer activity of curcumin has been known for a long time, its therapeutic potential is severely limited due to poor solubility. Therefore, it is of significance to investigate and develop wax-based SLNs for efficient drug loading and the use of curcumin with microbial biofilms.

In this study, the SLNs were fabricated with Chinese white wax. Initially, the formulation process of the empty Chinese white wax SLNs (cwSLNs) was optimized. Using the optimized formulation, the curcumin loaded Chinese white wax SLNs (Cur-cwSLNs) were prepared and characterized. Finally, the antibacterial activity and the reduced ability to form *Staphylococcus aureus* biofilms on this Chinese white wax-based Cur-cwSLNs were studied in vitro. This work firstly offered a novel solid lipid nano-carrier prepared with Chinese white wax for efficient curcumin loading, and secondly achieved effective results for inhibiting the formation of *S. aureus* biofilms.

## 2. Materials and Methods

### 2.1. Materials

Chinese white wax was from Cross-century Biotechnology Co., Ltd. (Suzhou, China). Tween 20 and lecithin were purchased from Sinopharm Chemical Reagent Co., Ltd. (Shanghai, China). All other chemicals used were either analytical or spectroscopic grade.

*Staphylococcus aureus* ATCC^®^12600^TM^ strain was maintained in nutrient agar as well as subculture in the laboratory, and it was revived and cultured at 37 °C in tryptic soy broth (TSB, Oxoid, Hampshire, UK). A 1 mL aliquot of overnight culture was inoculated into 100 mL fresh TSB, incubated at 37 °C with rotation, and grown to achieve 1 × 10^8^ CFU/mL.

### 2.2. Preparation of Empty Chinese White Wax SLNs (cwSLNs)

**Figure 1:**
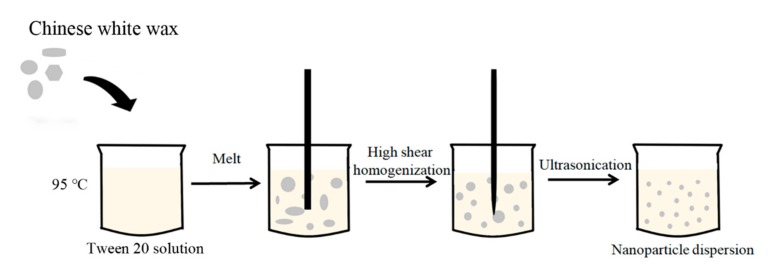
Schematic representation of Chinese white wax solid lipid nanoparticle (cwSLN) formation using the high shear homogenization and ultrasound method.

The cwSLNs were prepared by high shear homogenization and ultrasound method (Figure 1). Briefly, solid lipid was melted and dispersed in the 1% (*v*/*v*) Tween 20 solution at 95 °C using a homogenizer (IKA, Staufen, Germany) at 15,000 rpm for 30 min. The obtained emulsion was ultrasonicated using a probe sonicator (Scientz, Ningbo, China). The sonification procedure was: Duration time =3 s per cycle, total cycles = 100, interval between cycles = 3 s, and power = 30%. The resulting nanoemulsion was cooled down to produce a nanoparticle dispersion.

#### 2.2.1. Formula Optimization of the cwSLNs

In order to obtain the formula of cwSLNs to reveal the one that could result in the most appropriate nanoparticle, a different formula was optimized from the original one, which was homogenization time (HT) = 30 min), sonication time (ST) = 6 min, Tween 20 surfactant concentration (SC) = 1% (*v*/*v*), lipid concentration (LC) = 1% (*w*/*v*), and lipid= Chinese white wax.

The optimization was carried out by varying:

(a) HT = (20, 30, and 40 min), while ST = 4 min, SC = 1% (*v*/*v*), and LC = 1% (*w*/*v*);
(b) ST = (6, 8, and 10 min), while HT = 40 min, SC = 3% (*v*/*v*), and LC = 1% (*w*/*v*);
(c) SC = (1%, 3%, and 6%, *v*/*v*), while HT = 40 min, ST = 10 min, and LC = 1% (*w*/*v*);
(d) LC = (0.5%, 1%, and 2%, *w*/*v*), while HT = 40 min, ST = 10 min, and SC = 3% (*v*/*v*).

In each group, cwSLNs were prepared following the homogenization and ultrasonication parameters described above.

#### 2.2.2. Preparation of Curcumin Loaded Chinese White Wax SLNs (Cur-cwSLNs)

Cur-cwSLNs were prepared by the high shear homogenization and ultrasound method optimized in Section 2.2.1. In brief, 0.066 g of Chinese white wax and 0.034 g of lecithin were dissolved in the 10 mL 3% (*v*/*v*) Tween 20 solution. The mixture heated at 95 °C and then subjected to homogenization, during which 0.01 g of curcumin was supplemented into this mixture. After homogenization, the dispersion was ultrasonicated by ultrasonicator. Then the nanoparticle dispersion was allowed to cool at room temperature.

### 2.3. Characterization of Chinese White Wax-Based SLNs

#### 2.3.1. Determination of Particle Size and Polydispersity

Particle sizes and polydispersity index (PDI) of all Chinese white wax-based SLNs were measured by using a Malvern Zetasizer Nano ZS (Malvern Instruments, Worcestershire, UK) at 25 °C. The SLNs were appropriately diluted with deionized water before subjecting them to the apparatus. 

#### 2.3.2. Zeta Potential Measurement

Zeta potential (ZP) of all Chinese white wax-based SLNs were determined by the measurement of the electrophoretic mobility using a Malvern Zetasizer Nano ZS. The field strength applied was 20 V cm^−1^. The SLNs were also diluted using ultra-purified water as stated above.

#### 2.3.3. Entrapment Efficiency

The entrapment efficiency (EE) was determined by measuring the concentration of unentrapped curcumin. Briefly, after Cur-cwSLNs suspension was freshly prepared, 1 mL of this suspension was centrifuged at 20,000 rpm in a cooling centrifuge at 4 °C for 20 min. Then, the supernatant was appropriately diluted with aqueous phase (containing 3% (*v*/*v*) Tween 20 to solubilize curcumin). The amount of drug left unentrapped in the aqueous phase was detected by using UV/Vis spectroscopy (TU 1810, Beijing, China) at 425 nm with a preestablished curcumin standard curve. The percent of entrapment for Cur-cwSLNs was determined using the following equation.

$$EE\;\left(\%\right)=\frac{\;\mathrm{Initial}\;\mathrm{amount}\;\mathrm{of}\;\mathrm{curcumin}\;\mathrm{added}\;-\;\mathrm{Unloaded}\;\mathrm{amount}\;\mathrm{of}\;\mathrm{curcumin}\;\mathrm{in}\;\mathrm{aqueous}\;\mathrm{phase}}{\mathrm{Initial}\;\mathrm{amount}\;\mathrm{of}\;\mathrm{curcumin}\;\mathrm{added}}\times \;100\%$$

#### 2.3.4. ABTS Assay

Radical scavenging activity was measured as previously described [16] but with some modifications. First, 10 mL K_2_S_2_O_8_ (2.6 mmol/L) and 50 mL 2,2′-azino-bis (3-ethylbenzothiazoline-6-sulphonic acid, ABTS) (7.4 mmol/L) were mixed in an amber bottle for 12–16 h. After that, the mixture was used as a work solution that was diluted with PBS (pH 7.4) to obtain an absorbance of 0.700 ± 0.020 at 734 nm by a UNIC UV-2100 spectrophotometer. After that, various concentrations of curcumin and Cur-cwSLNs were added and reacted with the ABTS solution for 10 min at 25 °C. Then, the absorbance was read at 734 nm. All the results were expressed as mg vitamin C equivalent antioxidant capacity per µL sample.

#### 2.3.5. Transmission Electron Microscopy (TEM)

TEM observations were performed after negative staining of all Chinese white wax-based SLNs with phosphotungstic acid. Briefly, the sample was mixed thoroughly with an equal volume of phosphotungstic acid solution. Afterwards the mixture was incubated for 5 min. Subsequently, a drop of this mixture was dropped onto carbon-coated grids with the excess drawn off by filter paper. Finally, the grids were dried and imaged by a transmission electron microscope (H-600 A, Tokyo, Japan).

#### 2.3.6. Differential Scanning Calorimetry (DSC)

DSC thermograms of the starting synthesis materials and Chinese white wax-based SLN samples were obtained after sample preparation. The thermograms were recorded by a DSC (TA Q2000, Delaware, CT, USA). For measurements, freeze-dried samples were placed on an aluminum pan and the thermal behavior determined in the range of 30–350 °C at a heating rate of 10 °C per minute. An empty sealed pan was used as reference.

#### 2.3.7. In Vitro Drug Release and Cytotoxicity

The in vitro release study of Cur-cwSLNs was performed in different pH conditions (pH 4.5 and 7.4). Generally, Cur-cwSLNs were suspended in 1 mL of phosphate buffer of pH 4.5 and 7.4 to reach the final concentration of 1 mg/mL, respectively. Then they were transferred into dialysis bags (molecular weight: 8000–14,000) and dialyzed against 50 mL corresponding phosphate buffer (pH 4.5 and 7.4) containing 2% Tween 20 in a 100 mL beaker at 37 °C under magnetic stirring at 100 rpm [17]. An aliquot of 1 mL was withdrawn at predetermined time intervals and replaced with an equal volume of fresh buffer. Curcumin released in the buffer was measured spectrophotometrically at OD_425nm_. The control nanoparticles formulated without curcumin were treated similarly and used as a control for measurements. The experiments were carried out in triplicate. The in vitro cytotoxicity of cwSLNs and Cur-cwSLNs were performed with L929 cells using the MTT assay as described previously [18]; the concentrations of cwSLNs and Cur-cwSLNs were set to be 1, 5, 10, 20, and 50 µg/mL.

### 2.4. In Vitro Anti-Biofilm Study

#### 2.4.1. Determination of the Minimum Inhibitory Concentration (MIC)

The minimum amount of curcumin and Cur-swSLNs required for inhibiting the growth of *S. aureus* ATCC^®^12600^TM^ strain after a definite period of incubation was estimated by performing the MIC test with the broth dilution method [19]. For this purpose, eight different concentrations, including 5, 15.625, 31.25, 62.5, 125, 250, 500, and 1000 µg/mL of both curcumin and Cur-cwSLNs, were tested against *S. aureus* ATCC^®^12600^TM^ strain.

#### 2.4.2. Effects of Cur-cwSLNs on Reducing the Formation of *S. aureus* Biofilm

An overnight culture of *S. aureus* ATCC^®^12600^TM^ was diluted to 1 × 10^6^ CFU/mL with the media containing curcumin and Cur-cwSLNs, at a concentration of 125 µg/mL, in 96-well microtiter plates. After incubating for 90 min at 37 °C without shaking, non-adherent bacterial cells were removed by washing with 200 µL of PBS, using pipettes, following by the addition of fresh media. Then, they were incubated for 48 h at 37 °C without shaking. After incubation, each well was washed three times with PBS buffer (200 µL) to remove unattached bacterial cells. Then, they were stained with 0.1% (*w*/*v*) crystal violet (Sinopharm, Beijing, China) solution for 30 min and washed again with PBS buffer. Then, 200 mL of 30% acetic acid (*v*/*v*) was added to each well for 15 min to extract the crystal violet retained by the cells. The OD_630nm_ was measured with a microplate reader to determine the amount of biofilm (RT-2100C, Rayto Life and Analytical Sciences Co., Ltd., Hamburg, Germany). All experiments were repeated six times.

#### 2.4.3. SEM Observation

The effects of Cur-cwSLNs on *S. aureus* ATCC^®^12600^TM^ biofilm adherence to medical material surfaces were evaluated using catheters (Polyvinyl chloride, Jianshi, China). The catheters (length, 0.6 cm; inner diameter, 0.16 mm; outer diameter, 0.23 mm) were sterilized and placed into wells of a 6-well microplate. After that, 6 mL of culture containing Cur-SLNs and curcumin solution (500 µg/mL) were added and incubated at 37 °C for 48 h without shaking. Then, plates were rinsed with PBS. For scanning electron microscope (SEM) observation, the catheters with biofilm were fixed in 5% (*v*/*v*) glutaraldehyde (Sinopharm, Beijing, China) in PBS solution overnight at 4 °C, and then subjected to serial dehydration with 70%, 80%, 95%, and 100% ethanol (Sinopharm, Beijing, China) for 20 min each. The dried biofilm was gold coated, and then examined with an SEM (Jeol Ltd., Akishima, Tokyo, Japan).

### 2.5. Statistical Analysis

All experiments were conducted in triplicate, and crystal violet staining was repeated six times. Results were expressed as mean ± standard deviation. Multiple range comparison and least significant difference (LSD) tests were used to perform the statistical evaluation of the data. Differences were verified to be statistically significant if the *p* value was less than 0.05.

## 3. Results and Discussion

### 3.1. Preparation and Characterization of Blank cwSLNs

In this work, SLNs were prepared with Chinese white wax for the first time by using the high shear homogenization and ultrasound method [20,21]. Meanwhile, the conditions for preparing the SLNs were also optimized. In Table 1, the effects of different process variables on average particle size, polydispersity index (PDI), and zeta potential (ZP) of the obtained cwSLNs were summarized.

It was noted that there were no significant differences in average particle size and PDI of SLNs with different homogenization times (HT). However, sonication time (ST) obviously influenced the PDI of obtained SLNs, although it only slightly reduced their average particle size. With an increase in ST, the size and PDI of particles decreased. A longer ST added more sonication energy to the cwSLNs so that they were better dispersed, leading to a reduced PDI [22]. As a result, the ST of 8 min was determined to be optimal. Subsequently, it was found that the surfactant concentration (SC) had a significant influence on the particle size and PDI of cwSLNs. The use of 3% surfactant resulted in the smallest particle size; therefore, it was considered the optimal concentration. Notably, the average particle size of SLNs was obviously affected by the lipid concentration (LC). As seen in Table 1, the use of 1% LC led to a minimum average particle size of 401.9 ± 21.3 nm for the obtained cwSLNs, whereas with 0.5% and 2% LC, the average size and PDI of the resulting SLNs was much larger than that obtained with 1% LC. It is possible that too low a concentration of Chinese wax cannot be dispersed effectively, but too high a concentration of Chinese wax can be too easily coalesced with other particles at high wax concentrations, which was in accordance with the SLNs prepared with the lipids of Houttuynia cordata extract [21] and paclitaxel [23].

From the results discussed above, the optimized formulation conditions were determined as follows: HT = 40 min, ST = 8 min, SC = 3% (*w*/*v*), and LC = 1% (*w*/*v*). Under optimal formulation conditions, the particle size, PDI, and zeta potential of cwSLNs were 401.9 ± 21.3 nm, 0.245 ± 0.018, and −25.9 ± 6.7 mV, respectively. The following cwSLNs were prepared according to these conditions, unless specially mentioned.

### 3.2. Characterization of Cur-cwSLNs

**Figure 2:**
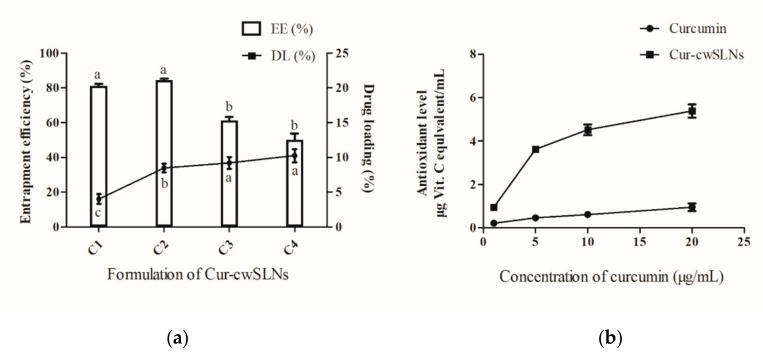
(**a**), The entrapment efficiency (EE) (%) and drug loading (DL) (%) of curcumin (Cur)-cwSLN with various formations. (**b**), Antioxidant levels between free curcumin and Cur-swSLNs at different concentrations. Data are presented as the mean ± SD, *n* = 3. ^a^^,b^, data with different superscript were significantly different (*p* < 0.05).

**Figure 3:**
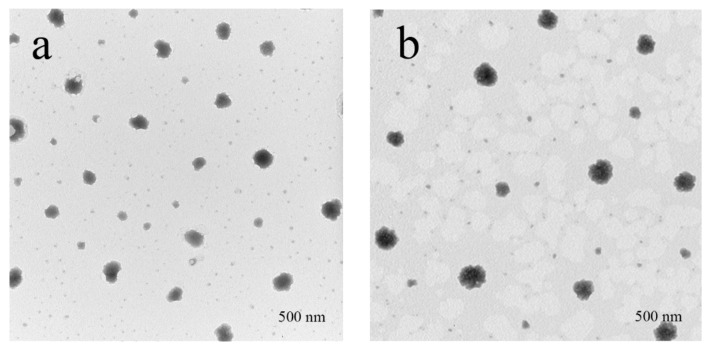
The TEM of the SLNs. (**a**) cwSLN; (**b**) Cur-cwSLN.

After curcumin was loaded into cwSLNs under the optimized conditions, the average particle size of Cur-cwSLNs varied from those of empty cwSLNs. It can be seen from Table 2 that the increased curcumin concentration led to slightly increased average particle size of Cur-cwSLNs. Subsequently, the entrapment efficiency and drug loading of each group of Cur-cwSLNs was determined. As seen in Figure 2b, 10% of the curcumin led to approximately 85% entrapment efficiency (EE) and 10% curcumin (drug) loading (DL), similar to that of the SLNs made from Imwitor 900 K [24], Compritol HD5 [24], and Trilaurin SLNs for loading curcumin [25]. These data indicated that the Chinese white wax was an acceptable new matrix candidate for preparing highly efficient curcumin loaded SLNs. Therefore, 10% curcumin was selected as the optimal concentration for preparing Cur-cwSLNs. Meanwhile, it was noted that the heating treatment that was used in the processing of Cur-cwSLNs might cause side effects to the stability and bioactivity of curcumin. However, the high entrapment efficiency (approximately 84.6%) indicated that these operations had no obvious effects in terms of disintegrating curcumin. Moreover, the bioactivity of the encapsulated curcumin was taken into consideration. The ABTS test (Figure 2) showed that the Cur-cwSLNs exhibited higher antioxidant activity than free curcumin. This was believed to be caused by the encapsulation of Chinese white wax after the formation of Cur-cwSLNs as protective shells, as with other reported SLNs [26]. Under the above condition, the average particle size was only slightly increased, to approximately 423.7 ± 23.2 nm. To verify this result, empty cwSLNs and drug loading Cur-cwSLNs were subjected to TEM observation. Figure 3 illustrates that the size of Cur-cwSLNs was indeed slightly enlarged compared with that of cwSLNs after the drug loading. Moreover, the particle size of Cur-cwSLNs was almost twice that of the reported curcumin loaded SLNs [24,25], which was attributed to the particularity of Chinese white wax.

**Figure 4:**
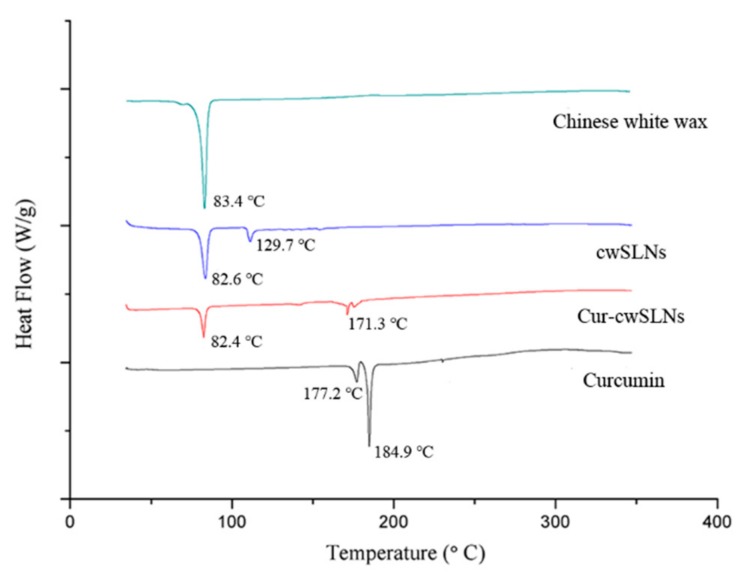
The differential scanning calorimetry (DSC) thermogram of Chinese white wax (**green**), empty Chinese white wax SLNs (cwSLNs, **blue**), Curcumin loaded cwSLNs (Cur-cwSLNs, **red**), and curcumin (**black**).

Subsequently, in order to assess the crystallinity of lipid matrices and the drug–lipid interactions, DSC was run for the pure Chinese white wax, cwSLN, Cur-cwSLN, and curcumin (Figure 4). The thermogram of pure Chinese wax exhibited an endothermic peak of 83.4 °C. In the DSC thermograms of empty cwSLN and Cur-cwSLNs, an endothermic peak was observed to be at 82.6 °C and 82.4 °C (blue and red curves), respectively. This depression of melting point could be attributed to the Kelvin effect, which was described by the Thomson equation [8]. The decreased melting point of empty cwSLNs and Cur-cwSLNs again indicated that the obtained particles were indeed at the nanometer scale [27]. Additionally, an increased polymorphic transition rate was due to the small particle size [28]. The Cur-cwSLNs did not show the melting peak as curcumin did at 184.9 °C (black curve), suggesting that curcumin was present in Cur-cwSLN in an amorphous form or a molecularly dispersed status [29]. 

### 3.3. In Vitro Curcumin Release

**Figure 5:**
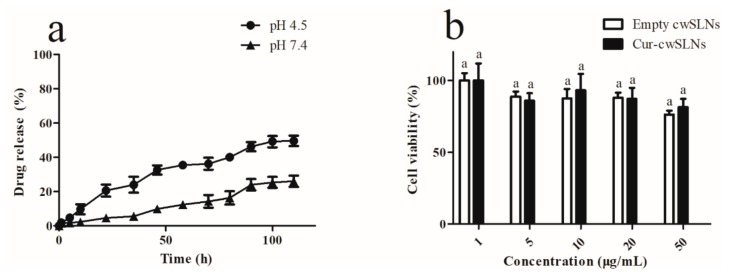
(**a**) The drug release of Cur-cwSLN in pH 7.4 and pH 4.5 over long periods. (**b**) The cytotoxicity of cwSLNs and Cur-cwSLNs in 24 h. Data are presented as the mean ± SD, *n* = 3. ^a^, data with same superscript were not significantly different (*p* > 0.05).

After the drug loading properties of the cwSLNs were addressed, the in vitro release of curcumin from the Cur-of inhibiting the formation of biofilms in human bodies. According to this different release profiles of Cur-cwSLNs in neutral and acidic pH, it appeared that the cwSLNs not only had a possibility to be applied in most parts of human bodies, where pH was maintained at near-neutral pH (7–9), but also were more suitable to be used in some special locations where the pH was acidic, cwSLNs was subsequently evaluated under both neutral and acidic pH conditions. As demonstrated in Figure 5a, the Cur-cwSLNs constantly had slow release of curcumin over 96 h in both environments of pH 7.4 and pH 4.5 without any burst release. The slow and constant release of curcumin was mainly due to the slow diffusion and dissolution of curcumin through the solid core of cwSLNs [30,31,32]. Notably, the released rate of curcumin from the Cur-cwSLNs was significantly elevated in pH 4.5 (about 46.2%) as compared to that in pH 7.4 (about 24.1%), which indicated that cwSLNs possessed the responsive property to acidic pH. Usually, the SLNs prepared with natural lipid, including fatty acid, glycerides, and waxes, lacked pH responsiveness [33]. The integration of artificially synthetic materials, such as 4-(2-Aminoethyl) morpholine [33] and (2-(2,4,6-trimethoxyphenyl)-1,3-dioxane-5,5-diyl)bis(methylene), into the fatty acids were used to grant the acidic pH responsiveness to the resulting SLNs. Nevertheless, it was found that the Chinese white wax-based SLNs naturally have an acidic pH responsive ability, indicating their unique features over the other lipids. As stated previously in this study, the Cur-cwSLNs were designed for the purpose of inhibiting the formation of biofilms in human bodies. According to this different release profiles of Cur-cwSLNs in neutral and acidic pH, it appeared that the cwSLNs not only had a possibility to be applied in most parts of human bodies, where pH was maintained at near-neutral pH (7–9), but also were more suitable to be used in some special locations where the pH was acidic, such as skin [34], vagina, inflammatory sites [35], and dental caries [36], etc. 

### 3.4. In Vitro Cytotoxicity

In order to investigate the cytotoxicity of cwSLNs and Cur-cwSLNs, various concentrations of SLNs were incubated with L929 cells for 24 h, and the cell viability was measured by MTT assay. As shown in Figure 5b, the cell viability was above 75% for all groups at concentrations, of cwSLNs and Cur-cwSLN, ranging from 1 to 50 µg/mL after incubation for 24 h. The low cytotoxicity of both cwSLNs and Cur-cwSLNs demonstrated that the cwSLNs were a new biosafe nanostructured lipid carrier.

### 3.5. In Vitro Anti-Biofilm Study

The antibacterial activity of Cur-cwSLNs and pure curcumin was evaluated against *S. aureus* with the minimum inhibitory concentration (MIC) test. The MIC of free curcumin was about 500 µg/mL, whereas the MIC of Cur-cwSLNs was 62.5 µg/mL. Thus, it was established that Cur-cwSLNs could promote anti *S. aureus* effects with a much lower dose. This was ascribed to the encapsulation and protection of curcumin by cwSLNs, so that the bioavailability of curcumin was greatly improved. Moreover, the nanoscale sizes of Cur-cwSLNs might have facilitated their adherence to the cell walls of *S. aureus* [37,38], which might have enhanced the diffusion of curcumin into the bacterial cell, reducing the required antibacterial dose [39,40]. 

**Figure 6:**
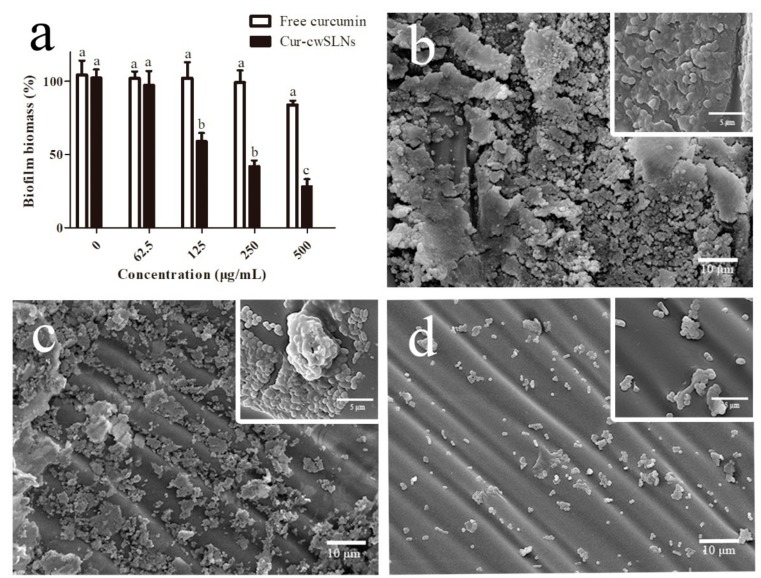
(**a**) Inhibiting effects for biofilms from *S. aureus* ATCC^®^12600^TM^ strain of free curcumin and Cur-cwSLNs. (**b**) SEM image of *S. aureus* biofilms, (**c**,**d**) SEM image of inhibitory effects of pure curcumin and Cur-cwSLNs against biofilms produced by *S. aureus*, respectively. Data are expressed as mean ± SD, *n* = 6. ^a,b,c^ in (a), data with different superscript were significantly different (*p* < 0.05).

Subsequently, colorimetric assays via crystal violet staining of biofilms were conducted to quantify the inhibition of biofilm formation by *S. aureus* ATCC^®^12600^TM^ after incubation with free curcumin and Cur-cwSLNs. As illustrated in Figure 6a, the Cur-cwSLNs significantly inhibited biofilm formation by *S. aureus* ATCC^®^12600^TM^ at the concentration of 125 µg/mL, whereas the free curcumin did not lead to obvious inhibitory effects, even when its concentration was increased to 500 µg/mL. The effects of biofilm inhibition were also confirmed by the SEM micrograph. As seen in Figure 6, the untreated *S. aureus* biofilms comprised a dense network of cells surrounded with vast amounts of exopolymer matrix (Figure 6b). As observed, individual curcumin had a weak effect on the formation of *S. aureus* biofilms (Figure 6c), whereas they were significantly reduced after treatment with Cur-cwSLNs (Figure 6d). This result demonstrated that *S. aureus* biofilms were much more effectively inhibited using Cur-cwSLNs rather than individual curcumin. From these in vitro assays, we confirmed that the Chinese white wax SLNs, with curcumin loading, could significantly interfere with and reduce the formation of *S. aureus* biofilms.

Curcumin reduced biofilm formation by inhibiting the genes involved in the initiation and quorum sensing of biofilms, as well as the down regulation of the virulence factors of biofilm forming bacteria [15]. However, bacterial biofilms were three-dimensional structures, in which cells were enmeshed in a substantial amount of EPS [41]. The dense and amphiphilic nature of the EPS matrix prevented the penetration of exogenous agents [42,43]. This was the reason the individual curcumin could not stop the formation of *S. aureus* ATCC^®^12600^TM^ biofilms (Figure 6c). Nevertheless, the nanoscale sizes of Cur-cwSLNs might aid their permeation through the EPS of *S. aureus* biofilms, and their sustained release property could guarantee consistent effects of released curcumin over a long period. Besides, the increased release rate of Cur-cwSLNs at acidic pH, which was also the microenvironment of bacterial biofilms [40,44], could enhance the inhibitory effects of curcumin against *S. aureus* ATCC^®^12600^TM^ biofilms. Therefore, the loading of curcumin in cwSLNs resulted in a satisfactory inhibition of biofilms produced by *S. aureus* ATCC^®^12600^TM^ (Figure 6d).

## 4. Conclusions

In closing, Chinese white wax solid lipid nanoparticles (cwSLNs) are highly efficient carriers for loading curcumin. With an optimized formula, the curcumin loading efficiency in cwSLNs can reach as much as 84.6%. The curcumin loaded cwSLNs (Cur-cwSLNs) possess a sustained drug release property and they are able to achieve a higher drug release rate under an acidic environment. Finally, Cur-cwSLNs are capable of inhibiting the formation of *S. aureus* biofilms. Thereby, the cwSLNs have the potential to serve as novel systems for the therapy of preventing the formation of biofilms produced by *S. aureus*, and it could provide guidelines for designing other drug-delivery systems to fight against various biofilms for human.

## Figures and Tables

**Table 1:** Effects of homogenization time (HT), sonication time (ST), surfactant concentration (SC), and lipid concentration (LC) on size, polydispersity index (PDI), and zeta potential of empty cwSLNs.

	Variables	Size (nm)	PDI	ZP (-mv)
HT (min)	20	/	/	/
	30	729.4 ± 23.7 ^b^	0.538 ± 0.023 ^a^	23.9 ± 4.3 ^a,b^
	40	583.9 ± 30.2 ^c,d^	0.467 ± 0.043 ^b^	27.4 ± 3.2 ^a^
ST (min)	4	626.6 ± 19.3 ^c^	0.428 ± 0.030 ^b,c^	25.7 ± 1.9 ^a,b^
	6	532.7 ± 22.5 ^d^	0.376 ± 0.037 ^c^	24.1 ± 5.8 ^a,b^
	8	458.3 ± 25.0 ^e^	0.337 ± 0.029 ^c^	27.8 ± 4.2 ^a^
SC (%, *w*/*v*)	1	664.6 ± 66.3 ^b,c^	0.361 ± 0.022 ^c^	19.2 ± 2.6 ^a,b^
	3	450.8 ± 34.6 ^e^	0.332 ± 0.031 ^c^	24.8 ± 4.8 ^a,b^
	6	498.3 ± 31.6 ^d,e^	0.406 ± 0.048 ^b,c^	25.9 ± 5.1 ^a^
LC (%, *w*/*v*)	0.5	950.7 ± 98.2 ^a^	0.553 ± 0. 023 ^a^	13.2 ± 8.2 ^b^
	1	401.9 ± 21.3 ^f^	0.245 ± 0.018 ^d^	13.2 ± 8.2 ^b^
	1.5	475.8 ± 55.9 ^d,e^	0.402 ± 0.021 ^b,c^	20.4 ± 4.2 ^a,b^

Results were given as mean ± SD; *n* = 3. /, date were unable to be determined; ^a,b,c,d,e,f^, data with different superscripts were significantly different (*p* < 0.05).

**Table 2:** Effects of the curcumin concentration on size and PDI.

Curcumin Concentration	Average Particle Size (nm)	PDI
5%	411.8 ± 17.9 ^b^	0.241 ± 0.082 ^a^
10%	423.7 ± 23.2 ^b^	0.310 ± 0.076 ^a^
15%	476.3 ± 28.5 ^a^	0.192 ± 0.063 ^a^
20%	498.5 ± 25.0 ^a^	0.218 ± 0.064 ^a^

Results were given as mean ± SD; *n* = 3. ^a^^,b^, data with different superscript were significantly different (*p* < 0.05).
